# Public Pharmacists' Association

**Published:** 1921-02-12

**Authors:** 


### Public Pharmacists' Association.

The Annual General Meeting of this Association was held at St. Bride Institute, London, on January 26, Mr. J. L. P. Hollingworth, F.L.S. (Victoria Hospital, Chelsea) presiding. A presentation was made to Mr. G. W. Gibson, Pharmacist, St. Pancras Hospital, London, who now retires from the Hon. Secretaryship after ten years' service. The present took the form of a handsome easy-chair, upholstered in corduroy. Mr. Hollingworth, who made the presentation, referred to the valuable services rendered to the Association by Mr. Gibson, and he was supported by several members present. Mr. L. Billington, Islington Infirmary, Highgate Hill, is the Hon. Secretary for 1921.

After the general business a keen discussion took place on the Draft Regulations under the Dangerous Drugs Act, now published. Mr. Arthur Jenkin explained the Regulations in detail, and pointed out how unworkable these were for hospitals and institutions. The one satisfactory point was noted that in the Draft Regulations pharmacists at hospitals are "authorised persons" so far as possession and supply of the drugs goes without obtaining a special licence. The Hon. Secretary was instructed to send a resolution of protest to the Home Office to the effect that the Draft Regulations were not suited for the conditions obtaining in public institutions.

